# Use of Cyclosporine in Uterine Transplantation

**DOI:** 10.1155/2012/134936

**Published:** 2011-11-10

**Authors:** Srdjan Saso, Karl Logan, Yazan Abdallah, Louay S. Louis, Sadaf Ghaem-Maghami, J. Richard Smith, Giuseppe Del Priore

**Affiliations:** ^1^Division of Surgery, Oncology, Reproductive Biology and Anaesthetics, Department of Surgery and Cancer, Institute of Reproductive and Developmental Biology, Imperial College London, Hammersmith Hospital Campus, Du Cane Road, London W12 0NN, UK; ^2^Gynaecological Oncology, West London Gynaecological Cancer Centre, Queen Charlotte's Hospital, Hammersmith Hospital Campus, Imperial College London, Du Cane Road, London W12 0NN, UK; ^3^Melvin and Bren Simon Cancer Center, Indianapolis, Indiana University School of Medicine, Simon Cancer Center, Indianapolis, IN 46202, USA

## Abstract

Uterine transplantation has been proposed as a possible solution to absolute uterine factor infertility untreatable by any other option. Since the first human attempt in 2000, various teams have tried to clarify which immunosuppressant would be most suitable for protecting the allogeneic uterine graft while posing a minimal risk to the fetus. Cyclosporine A (CsA) is an immunosuppressant widely used by transplant recipients. It is currently being tested as a potential immunosuppressant to be used during UTn. Its effect on the mother and fetus and its influence upon the graft during pregnancy have been of major concern. We review the role of CsA in UTn and its effect on pregnant transplant recipients and their offspring.

## 1. Background

Uterine transplantation (UTn) has been proposed as a possible solution to absolute uterine factor infertility (AUFI) untreatable by any other option [1]. The inability to experience a pregnancy, give birth, and bring up a child because of infertility issues may be one of the most traumatic and devastating situations to affect a woman or couple, with the capacity to have a severely detrimental effect on the quality of life of both parties [2–4]. The term “infertile” is an all-encompassing term and includes women with AUFI. Causes which render women “unconditionally infertile” are either congenital, namely, Müllerian duct anomalies, or acquired causes (such as leiomyomata, radiation damage, intrauterine adhesions, or premenopausal hysterectomy for obstetric bleeding or cervical/endometrial cancer).

Tremendous advances have been made during the last decades in the fields of transplantation and reproductive medicine, in particular, the first reports of successful transplantation of a solid organ, the kidney [5, 6], and live birth after IVF [7]. Transplantation surgery today includes types of organ/tissue transplantation that will enhance the quality-of-life as exemplified by transplantation of the hand [8], the abdominal wall [9], the larynx [10], and the face [11], with the aim to add UTn to the list.

UTn was first performed in a human in 2000 on a 26-year-old female who had previously lost her uterus as a result of postpartum haemorrhage. The event granted much needed impetus for research into UTn, which since then has been a slow, methodical process within an animal setting involving multiple institutions and disciplines over several continents. Research has focused on several important areas, mainly surgical, immunological, and reproductive aspects. The second UTn attempt in a human model is anticipated in the not-too-distant future.

A major obstacle to UTn remains immunological rejection of the transplanted graft. The process of rejection in allogeneic UTn was first described by two studies in 1969 [12, 13], the decade in which UTn research was first published. At that time, UTn commonly involved *en bloc* autotransplantation of a combination of the different reproductive organs: uterus, fallopian tubes, and ovaries. Since then, assisted reproductive technologies such as *in vitro* fertilization (IVF) have addressed many of the causes of infertility. However, for women who suffer from AUFI, surrogacy, adoption, or lifetime infertility remains the only option. The hope is that, with continued advances in transplantation surgery and control of tissue rejection, UTn can bring an end to AUFI by allowing a select group of women to become mothers [14].

## 2. Transplant Surgery

The treatment of choice for certain end-stage organ diseases, such as kidney and liver failure, is organ transplantation. Since 1956 and Joseph Murray's first successful solid-organ transplant, involving the transfer of a kidney between genetically identical twins, this field has progressed rapidly because of considerable improvements in two vital areas: surgical methodology and immunology. With respect to the latter, it is important to recognise that without full understanding of the risks posed by the rejection process and subsequent development of immunosuppressants, transplantation as we understand it today would not be possible. It would certainly involve a smaller group of possible donors (only syngeneic), resulting in a greatly reduced number of potential beneficiaries.

Research of graft rejection between two allogeneically different individuals resulted in the development of immunosuppressive agents in the 1960s. 5-mercaptopurine/azathioprine, in combination with corticosteroids, was the first to be marketed for use. Next came the discovery of the calcineurin inhibitor cyclosporine A (CsA) in the late 1970s [15]. These drugs, especially CsA, represented at the time a widely sought *panacea* and caused a rapid advancement in clinical organ transplantation.

The risks posed by immunosuppressants are well known, and the potential teratogenicity of certain agents still poses a considerable threat. At the time of the first healthy birth to a transplant recipient in 1958 [16], pregnancy was not recommended by both transplant surgeons and obstetricians [17]. However, approximately 14,000 births (more since this result was first published in 2002) among transplant women have been reported worldwide [18], resulting in a change of opinion, whereby restored fertility is now a recognized desirable outcome following transplantation [19, 20].

## 3. Immunosuppression in General

Sir Peter Brian Medawar first introduced the idea of immunosuppressants as possible agents that could be used to prevent and treat allograft rejection. Currently short-term effects have been very positive but chronic rejection is still a major issue and long-term use has its drawbacks. These are mainly related to nonspecific suppression of the host immune system, which may cause any of the following: bacterial and viral infections, nephrotoxicity and neurotoxicity with chronic renal failure, newly diagnosed diabetes mellitus, hyperlipidaemia, leucopoenia, and even cancer [15, 21].

Deciding on the type, dose, and monitoring of immunosuppressants prescribed is best made within a multidisciplinary team setting including an obstetrician, so as to maximize the efficacy and minimize potential toxicity [19]. As immunosuppressants can cross the human placental barrier and enter the fetal circulation, possibly affecting the immune system of the fetus, obstetric input is paramount [22]. Which immunosuppressant is administered depends very much on the age and comorbidities of the patient, type of transplanted organ, length of time that the patient is expected to live postoperatively, and the transplant centre. Drug combinations and immunosuppressant protocols vary considerably between centres. 

Medications used to suppress immunologic activity are chosen based on the stage of the transplant process. In the induction phase, intravenous immunoglobulin is usually combined with a cornerstone immunosuppressant such as a cytokine modulator (CsA), purine-related analogue (azathioprine), and a purine synthesis inhibitor (mycophenolate mofetil). CsA or tacrolimus are then usually continued as “maintenance therapy,” in combination with glucocorticoids, mycophenolate mofetil/sodium, or azathioprine. In addition, alkylating agents (cyclophosphamide and chlorambucil) and biologic response modifiers (TNF antagonists) can be used [15, 24]. 

The first record of the use of immunosuppressants in pregnancy was in 1967 by Board et al. This team used azathioprine and prednisolone to prevent allogeneic rejection in a kidney recipient [25]. The risk that immunosuppressants pose to both the mother and fetus, with respect to potential birth defects, needs to be fully investigated and understood. This is especially important as the number of female transplant recipients of child-bearing age is increasing. Furthermore, problems related to thalidomide exposure in the 1960s clearly portray the devastating consequences of the release of unknown drug into the general population [26].  Table 1 summarises the risk categories of commonly used immunosuppressants, including CsA. The level of risk is dependent on whether an animal or human is receiving the immunosuppressant as well as the dose and route of administration of the immunosuppressant. Fetal risk ranges from severe abnormalities related to structural development that are diagnosable either at any stage of pregnancy or only in the third trimester, to those that are only subtle and apparent after delivery [27].

## 4. Cyclosporine

CsA is a cyclic nonribosomal decapeptide derived from the fungus, *Tolypocladium inflatum*, first isolated in Norway in 1969. It was first used to successfully prevent kidney [28] and liver transplant rejection [29], with official approval for CsA use arriving in 1983. Apart from transplant medicine, CsA is also used in a number of autoimmune diseases: dermatological-psoriasis; severe atopic dermatitis; pyoderma gangrenosum; rheumatologic-rheumatoid arthritis and gastrointestinal such as ulcerative colitis and Crohn's disease.

The main role of CsA is to interfere with signaling pathways important for the clonal expansion of immunocompetent lymphocytes. It does this by forming complexes through binding to an intracellular cytosolic protein called cyclophilin. The CsA-cyclophilin complex inhibits the phosphatase activity of a calcium-activated enzyme called calcineurin. Calcineurin is responsible for transmitting signals from the T-cell receptor to the nucleus. It is activated following the rise of intracellular Ca^2+^ levels in response to T-cell receptor binding. Calcineurin then dephosphorylates the transcription factor NF-AT (nuclear factor of activated T cells) in the cytoplasm, allowing for its migration to the nucleus which leads to the induction of transcription of genes coding for IL-2, CD40 ligand, and Fas ligand [30]. Therefore, by inhibiting the function of calcineurin, the CsA-cyclophilin complex manages to block clonal T-cell proliferation in response to the host immune system recognizing a specific antigen as being foreign. This results in reduced function of effector T cells [30]. CsA also acts on other immunopotent cells and has a large variety of other immunological effects (Table 2) [23].

## 5. Use of Cyclosporine in Uterine Transplantation

### 5.1. Human Attempt

Research into UTn restarted before its afore-mentioned milestone in 2000, that is, the first human attempt. This attempt may have been premature, with little characterization or understanding of the rejection response prior to the operation. CsA was administered 6 hours prior to surgery and postoperatively (4 mg/kg/body weight divided into two doses to assure a serum trough level of 200 ng) along with azathioprine, prednisolone and a boost by antithymocyte globulin. CD^+^4/CD^+^8 ratio in blood and Doppler of uterine blood flow were the only techniques employed to monitor possible rejection patterns. The first rejection episode occurred on the ninth postoperative day and the patient was treated by increasing the oral doses of CsA and azathioprine and administering an intravenous dose of prednisolone. The rejection resolved after 2 days but only after antithymocytic globulin was given. On the 99th day, following symptoms suggestive of uterine infarction, a hysterectomy was performed. Histopathologic examination confirmed thrombosis of uterine vessels, and, interestingly, there was no signs of rejection with apparent viability of both tubes [31].

### 5.2. Animal Models

To date, CsA has not been used as an immunosuppressant in the few cases where the primary objective was to bring about pregnancy following UTn. 4 cases involved a syngeneic model which removed the need for an immunosuppressant [32–35]. In October of the last year, an allogeneic uterine transplant reported resorbed pregnancies in 4 out of 5 pregnant rats, with only tacrolimus used as an immunosuppressant [36]. 

However, CsA has been tried as an immunosuppressant in UTn studies where achieving pregnancy was not the primary objective. Described below are all studies where CsA was administered as an immunosuppressant in UTn. Its first recorded use was in 1986 when Confino et al. described unilateral nonvascular UTn in 18 rabbit models. Out of those, 6 were allogeneic transplants with administration of CsA. Three rabbits developed pelvic abscesses while in three other rabbits both the endometrial and myometrial layers survived for four weeks [37]. Brännström group went on to describe the acute rejection response in a mouse model, with signs of rejection from day 2 to day 5, represented by increased density of CD3+ T cells in the myometrium and endometrium, and full rejection with massive necrosis and fibrosis by day 28 [38]. Having demonstrated that predominately neutrophils, macrophages, and CD^+^4/CD^+^8 T cells were responsible for acute rejection [39], CsA was first tried as a potential immunosuppressant in a mouse UTn model. 5 mice acted as control (no CsA given) and 5 received either 10 or 20 mg/kg/day of CsA. As expected the extent of necrosis demonstrated on histology was decreased in the CsA mice. Apoptosis and inflammation were less prominent in grafts taken from the recipient mice that had received a higher CsA dose. Therefore, the authors concluded that administration of CsA can evidently delay the progress of rejection of grafted uteri. However rather surprisingly, T-cell infiltration was not suppressed with the CD8+ count significantly higher in the two allogeneic groups receiving CsA compared with the control group [40]. This outcome may be explained by CsA inhibition of activation-induced cell death (AICD) of cytotoxic T cells by major histocompatibility complex-peptide complexes [40, 41]. 

CsA has also been tried in a large-animal model. A single-agent CsA was effective for maintaining a uterus transplant in several nonhuman primate models. Clinical signs, serum analysis, and ultrasound examination of the uterus and vascular flow through the anastomosis were all used to monitor rejection and graft viability. In the first, the target CsA trough was 150 ng/mL, with a similar dose response to other solid organ transplants (5 mg/kg/day; dose mean: 15.5 ng/mL; 8 mg/kg/day mean: 191.8 *±* 59.3 ng/mL). This proved enough to prevent acute rejection in the immediate posttransplant period. However, during the second postoperative month, the following symptoms were reported: recipient weight loss and pelvic oedema. An apparent rejection episode was managed with additional immunosuppressants, high-dose steroids, and tacrolimus. No further rejection episodes occurred after that [1, 42]. The second model also revealed satisfactory results with CsA use. It was the only immunosuppressant administered with levels monitored and adjusted appropriately. On examination of the uterus a year after UTn, minimal signs of rejection were revealed with intact anastomosis [1, 43]. 

CsA and tacrolimus were administered to the same sheep model with successful results [44]. Initially, tacrolimus was given for 12 days intravenously, followed by CsA only orally. The uterus and ovary were harvested en bloc, flushed *ex vivo*, and transplanted in an orthotopic position by vascular anastomosis, either between individuals (allotransplantation, *n* = 9) or back to the same animal (autotransplantation, *n* = 7). Immunosuppression after allotransplantation consisted of administration of intravenous CsA (2–5 mg/kg) or oral tacrolimus (0.3–0.5 mg/kg) for 3 weeks, and the animals were then examined and euthanized. Autotransplanted ewes were evaluated at 7 weeks and then introduced to a ram (*n* = 5) for assessment of transplant function. In allotransplanted animals the ovary and uterus showed normal appearance in 33% (2 of 6) of CsA-treated and 66% (2 of 3) of tacrolimus-treated ewes at 3 weeks after transfusion. Histology analysis of these confirmed normal appearance. Analysis of uteri that diverged from normal gross appearance showed different degrees of inflammation, infiltration of leukocytes and necrosis. In the autotransplanted group, two animals were sacrificed 7 weeks after transplantation showing normal uteri and ovaries. Vaginal examination and analysis of serum progesterone confirmed satisfactory healing in the remaining 5 animals before a ram was introduced. Four ewes mated and three conceived, bearing one twin and two singleton pregnancies with normal foeti at termination on day 90 (*n* = 1) and day 140 (*n* = 2) of pregnancy. In conclusion, a model for UTn has been developed in the ewe with results indicating that immunosuppression by tacrolimus, rather than cyclosporine, is to be preferred in this model [44].

Furthermore, Avison et al. used CsA in a swine model. Ten transplants were performed, with 5 animals alive and healthy at the end of followup (0.5–12 months). Immunosuppression given was as follows: 10–20 mg/day of methylprednisolone for a month and oral CsA (10 mg/kg/day) as maintenance. Rejection episodes during the 2nd and 3rd months after transplantation were treated successfully with increased doses of oral CsA [45].

The above findings suggest that CsA administered at higher doses or used in combination with another type of immunosuppressant, as is the case in other organ transplants (kidney, liver, and heart), would be an ideal way of preventing UTn rejection. The effects of immunosuppressants on the maternal immune system have been well documented, and, therefore, viral serology for cytomegalovirus as well as microbiological cultures of vaginal smears repeated monthly may be helpful. Levels of CsA and other immunosuppressive drugs in the blood should be monitored, thus permitting adjustments of drug dose relative to graft function and physiological changes of pregnancy. 

Importantly, the risks to mother and fetus will be not different from those faced by renal, hepatic, or cardiac transplant patients undergoing pregnancy. All UTn patients should be managed within a multidisciplinary team setting, including a transplant immunologist. The care pathway would be very similar to any pregnant patient with a transplanted organ [46]. Visual inspection of the transplanted cervix would of course likely provide clinical clues of the graft's condition. In order to create a controlled environment during the labour process, a Caesarean section will be advisable. Removal of the transplanted uterus can be performed at the time of the Caesarean.

## 6. Cyclosporine and Pregnancy

For CsA to become the immunosuppressant of choice in UTn, its role in pregnancy and, therefore, its effect on both mother and fetus would have to be better understood. We describe below the different ways in which CsA can impact during the course of a pregnancy.

### 6.1. Correct Dosing-Graft Acceptance versus Toxicity

The aim of organ transplantation is to cure or at least reverse end-stage organ failure. Severe forms of both kidney and liver disease are associated with infertility. A significant proportion of all kidney and liver end-organ (thought to be higher than 50%) damage sufferers experience menstrual cycle-associated pathology, ranging from amenorrhoea to dysmenorrhoea and irregular cycles [47]. It has been demonstrated that following successful solid organ transplantation, as the graft begins to slowly function, normal endocrine and fertility function returns to the patient, allowing her to attempt pregnancy [47, 48]. Both renal and liver transplanted women report a restoration of their ovarian and menstrual function within an average of six to ten months following transplantation [49–51]. The hypothalamo-pituitary-ovarian axis in a transplant patient is thought to be functioning as normally as it would in a nontransplant female, with serum gonadotrophin and prolactin levels very similar in the two groups [49]. Higher levels of oestradiol are reported in renal graft patients in comparison to nontransplant women [52]. Therefore, restoration of fertility is often used as a variable to assess the function of the transplanted graft, with the two outcomes tending to mirror each other [47, 53].

Data on pregnancy outcomes of transplant patients is derived from voluntary registries, case reports, and retrospective centre studies. Davison and Baylis have summarised the outcomes of 2040 live births among female solid organ transplant recipients recorded in three major registries (the European Dialysis and Transplantation Association Registry, the UK Transplant Pregnancy Registry, and the National Transplantation Pregnancy Registry (NTPR) in the USA) [18]. Overall conclusions, particularly when analysing the NTPR data, indicate a higher incidence of maternal hypertension and preeclampsia in transplanted patients, with the number varying with the organ transplanted [54]. NTPR has reported hypertension rates of 47–73% in pregnant kidney-transplant recipients, a much higher rate than those demonstrated in pregnant women who have received liver, heart, or lung transplants. CsA is known to induce hypertension, and its more frequent use in kidney transplantation may explain such a high percentage [18]. Data derived from Radomski et al. [55] and Armenti et al. [56–58] revealed a hypertension incidence increase from 52% to 56–63% when nonemulsified CsA was administered and 68–73% when emulsified CsA (Neoral) was used. A similar picture is found with preeclampsia whose incidence varies from 25 to 45% with CsA use [55–58], with a third of all pregnant women receiving kidney or pancreas-kidney transplants reporting this diagnosis and only 25% of liver, heart, or lung recipients [18, 46, 52, 59]. 

The accuracy with which preeclampsia is diagnosed is open to debate. The two main markers of preeclampsia, hypertension and proteinuria, are found in many transplant patients (especially kidney) prior to pregnancy [17]. Furthermore, CsA increases uric acid levels, a blood marker for diagnosing preeclampsia, and therefore causes it to be less reliable [18, 60]. Both hypertension and preeclampsia must be either prevented or if already present controlled adequately. Indirectly, they lead to premature membrane rupture and subsequent preterm delivery and low birth weight [56, 57, 60]. This may explain why up to half of all pregnancies in transplant recipients, especially with renal grafts, end in preterm delivery [18, 46, 61, 62].

Administering the appropriate dose of CsA remains a challenge. A dose must be “low enough” not to bring about any toxicity within the mother or the neonate but at the same time “high enough” so as not to result in organ rejection. With respect to CsA, there may be no perfect dose but a “trial and error” approach which accepts the considerable variation in circulating drug levels associated with the increase in a pregnant woman's extracellular volume and altered pharmacokinetics [63]. Thomas et al. assessed the effects of pregnancy on CsA levels in six renal allograft patients and found that, after adjusting for dose, five of the six patients had declines in CsA level during pregnancy [63]. Frustratingly, there is very limited evidence, especially when searching for case-control studies of immunosuppressive levels in pregnant transplant women, to determine whether a drop in the blood concentration of an immunosuppressant below the prepregnancy level invariably leads to rejection [18]. Donaldson et al. presented a case of a pregnancy after bilateral lung transplantation. CsA levels dropped during the pregnancy which was complicated by acute and chronic allograft rejection, resulting in irreversible loss of lung function [64]. Furthermore, according to NTPR outcomes, pregnant kidney-transplant recipients who took higher doses of CsA before and during pregnancy maintained normal graft function in comparison to those patients who had smaller doses [18, 65].

Long-term data following long-term CsA use with regards to allograft loss, maternal survival after pregnancy (due to an increased risk of viral diseases and neoplasm secondary to immunosuppressants [66]), and offspring outcomes of transplant recipients is rather limited. Long-term outcomes focusing on physical and mental development of offspring and immunological and oncological pathologies later in life are necessary [46]. The comparison of pregnancy outcomes in a single group of women before and after transplantation can be of use. Currently, only one study by Källén et al. has carried out such work. It concluded that despite an initial increased risk for preeclampsia, growth restriction, preterm birth, and the risk of miscarriage, the odds of these 4 outcomes were all statistically similar before and after transplantation [67]. In fact, smoking and mother's comorbidity were the single most important determinants of fetal and neonatal well-being.

### 6.2. CsA and Toxicity during Pregnancy

The use of CsA throughout pregnancy exposes the fetus to potential fetotoxic and teratogenic agents. CsA, like other immunosuppressants, is required during the entire gestational period, with maintenance of appropriate CsA dose. It must not be reduced without reason or even worse, believing that natural nonspecific maternal immunosuppression can prevent allogeneic graft rejection. Any random reduction in CsA dosing may lead to rejection of transplanted organs, with two maternal deaths reported as due to discontinuation of immunosuppressive medications during pregnancy [68]. What makes this issue particularly challenging is the fact that such effects, if any, of CsA on the neonate with regards to phenotype and growth may be difficult to determine and may not be obvious at birth. In addition, underling maternal comorbidity, together with coexisting use of other types of immunosuppression and/or general medication, may act as a confounding factor and can therefore confuse the issue of whether it is the actual immunosuppressant responsible for the maternal or fetal effects [18]. 

CsA-related toxicity is a result of two important characteristics. First, the molecular targets of calcineurin inhibitors such as CsA are found in other cells types, which means that CsA may act on other tissues [23]. This explains why CsA is particularly toxic to both maternal and fetal renal tissue [69]. Intrauterine CsA exposure at each stage of development can induce permanent nephron and hepatic damage in the offspring. Fein et al. injected 30 mg/kg body weight of CsA into pregnant mice on days 6–8 or 10–12 of gestation. This dose did not raise the maternal mortality rate but histological examination revealed pathological alterations in the maternal thymus, liver, kidney, and spleen. Interestingly, most changes had disappeared by 1 week following the last injection. With respect to pregnancy, CsA reduced the number of viable embryos and increased the number of embryos resorbed. Organogenesis was not affected by the drug but CsA had an apparent embryotoxic effect [70]. 

Two studies by Tendron-Franzin et al. evaluated the impact of CsA on embryonic renal development in a rabbit model [71, 72]. Initially, twenty-one pregnant rabbits were injected with 10 mg/kg body weight of CsA for 5 days, either from days 14–18 or days 20–24 of gestation. The latter group demonstrated a reduced number of living pups, which were also growth-retarded. The former group exhibited normal fetal growth, and blood concentrations of CsA matched human data. Examinations of kidneys at birth suggested nephron mass and number reduction by 25 and 33% in both groups. Despite compensatory adaptation of the existing nephrons, renal function could face chronic problems because of the presence of segmental glomerular sclerosis [71]. The second study was also conducted in a rabbit model to assess the long-term systemic and renal effects of a CsA-induced (10 mg/kg body weight injected) nephron reduction. CsA intrauterine exposure led to (a) permanent nephron deficit, (b) glomerular, tubular and intrarenal haemodynamic dysfunction, (c) enlarged kidneys with numerous tubular and glomerular lesions, and (d) an endothelin-dependent systemic hypertension that worsened with age. This resulted in systemic hypertension and progressive chronic renal insufficiency in adulthood, suggesting that infants who are born to mothers treated with CsA during pregnancy must have long-term surveillance of kidney function in the form of blood tests and imaging [72]. For comparative purposes, we should emphasise that the dosages of CsA given in the animal models are applicable to those used in humans. CsA dosage depends on whether the drug is used at the induction stage or maintenance stage as well as on the institution and country. In the UK, adult dosage for induction therapy varies from 10 to 20 mg/kg.

In humans, the story is rather different with no similar effects identified [73, 74]. Shaheen et al. [75] and Giudice et al. [76] investigated renal function with the latter using inulin clearance, para-aminohippuric acid clearance, microalbuminuria, and electrolyte reabsorption rate in the offspring of transplant patients from birth to the age of seven. They concluded that, in children born to transplanted women taking CsA, renal function develops normally despite prolonged exposure *in utero*.

CsA can cross the placenta and thus enter fetal circulation [77], easily detected in the placenta, amniotic fluid as well as fetal tissues [78]. The level of CsA in fetal blood tends to be half the CsA level in the mother [75, 79] but even this level is enough to pose significant immunosuppression to the fetus [77]. Higher levels in the placenta and umbilical cord than in maternal blood have been recorded [18, 77, 79]. According to the Food and Drug Administration (FDA), CsA belongs to the Category C designation which indicates that human risk cannot be ruled out because studies in humans are lacking and studies in animals are either positive for risk or lacking all together. Until now, no consistent congenital malformations in the offspring of rodents exposed to CsA during pregnancy have been noted; however, a small number of reports have recorded: cataracts, growth delay, and fetotoxic effects at high doses [69, 80, 81]. 

There have been isolated reports of birth defects in humans but because of such small numbers and inconsistent patterns of defects it is difficult to conclude whether the incidence is secondary to CsA exposure or another factor [82]. The prevalence of major structural malformations in pregnant women without genetic history or disease is around 3 percent [17] which was approximately the same as the figure reported by NTPR (4-5 percent) [58]. A meta-analysis of 15 studies by Bar et al. concluded that CsA does not appear to be a major human teratogen but may be associated with increased rates of prematurity [83].

### 6.3. Cyclosporine and Fetal Immune Profile

The primary target of CsA in transplant recipients is the host immune system. As discussed above, apart from downgrading the maternal immune system, CsA manages to also enter fetal circulation during pregnancy. One would assume that this would result in CsA interrupting normal fetal T-cell development. However, only a limited number of studies have included the fetal and neonatal immune system as an end-point [18]. They suggest that immunosuppressants can have a profound effect on the development of an immune system. Heeg et al. exposed pregnant mice to a calcineurin inhibitor (CsA and tacrolimus) and, by doing so, brought about dysfunctional T-cell reactivity and prevented the generation of single positive mature T cells in newborn mice. Drugs were administered during the final third of the gestational period which is an important time for immune development. Also noticeable was the formation of hypoplastic peripheral lymphatic organs [84, 85]. Another similar study involving a rat model showed a series of immune perturbations including a decreased delayed type hypersensitivity response, splenic B cell number, and functional impairment during postnatal maturation [86]. 

Immunologic studies conducted in human neonates have so far been inconclusive. Some have reported normal immunologic function in infants exposed to immunosuppressants *in utero* [87, 88]. Another study of continuous exposure of CsA *in utero* in six infants born to female kidney transplant recipients demonstrated a seemingly impaired T-, B-, and NK-cell development and/or maturation (reduction in numbers and expression of CD25 and HLA-DR on T cells and CD5 on B cells), with most effects still apparent at one year [89]. Takhaashi et al. investigated the lymphocyte subpopulations in cord blood of six newborn infants born to mothers following renal transplantation. All were receiving CsA, as well as azathioprine and methylprednisolone. The number of B cells and the percentage of B cell in total mononuclear cells were significantly lower in these infants at one and three months of age, with no significant decrease between numbers of CD2+, CD4+, or CD8+ cells. This may suggest that the B-cell line is more sensitive to immunosuppressants *in utero* than the T-cell line [90].

Another threat posed by CsA and immunosuppression of the fetal immune system in general is a risk of autoimmune disease in the offspring of transplant recipients [88, 91]. In a particular study, CsA was administered to pregnant mice to study the effects of passively transferred CsA on the developing immune system which clearly altered the developing immune system. Embryos were partially depleted of CD4+ CD8− cells and 11 of 50 offspring born to CsA-treated mothers developed significant levels of IgG autoantibodies to gastric antigens. Two animals developed an extensive mononuclear cell infiltrate in the gastric mucosa resembling autoimmune gastritis [92]. However, in the Motherisk Program in Canada, the incidence of autoimmune diseases in the offspring of mothers with kidney transplants included one child with insulin-dependent diabetes mellitus and two children with asthma. These rates mimic the expected rates of the two disorders among the general population [93]. More studies are required for definitive conclusions.

### 6.4. Cyclosporine and Fetal Reproductive Profile

Part of the decision making process as to which immunosuppressant should be used depends on the effect of that immunosuppressant on pregnancy specific hormones. Groth et al. published the first study assessing reproductive health in animals exposed to immunosuppressive drugs (CsA) *in utero*. They also investigated the effect of CsA on pregnancy and fetal development [94]. Their results demonstrated that direct maternal and *in utero *exposure to high doses of CsA reduced implantation rates, fetal survival, and adolescent growth but did not affect offspring fertility. This correlation was dose dependent, with higher doses exhibiting a more pronounced negative effect. The reduced implantation rates have been reported in previous similar experiments involving murine models [69, 70]. In a particular study by Mason et al., female Lewis rats were given 10–25 mg/kg/day of CsA from the time of mating to 20 days after coitus. Higher CsA doses caused a powerful fetotoxic effect, resulting in a high incidence of fetal mortality or miscarriage, suggesting that CsA can affect the endometrium or decidua in a negative way [81]. In fact there is evidence which reveals a possible link between CsA and suboptimal ovarian function, as a result of hindrance to sex steroid secretion at the time of ovulation [95, 96]. This in turn leads to issues with conception which tends to occur at the time of ovulation. The endometrial layer depends on the sex steroids for its maintenance, and, if their secretion is impaired, the lining is also dysfunctional. As a result conception is impaired, an effect even more pronounced as a result of the role CsA plays in both T-cell and NK-cell activation [97].

Finally, it is worth noting that the effect of CsA upon the fetus continues after delivery if the mother breastfeeds. Data regarding breast feeding by mothers taking immunosuppressive medication is still relatively sparse. According to The American Academy of Paediatrics, steroids such as prednisolone pose no risk, whereas breast feeding by mothers taking CsA is actively discouraged. No recommendations regarding azathioprine or tacrolimus exist in the literature [98]. Measuring CsA levels in breast milk is currently not a recognised tool as levels vary greatly between different transplant recipients, from undetectable to equal to those in the maternal serum. Flechner et al. reported a term pregnancy in a CsA and prednisone-treated female cadaveric renal allograft recipient. A male child, small for gestational age at 2370 g, was born at 38 weeks of gestation with neither congenital anomalies nor nephrotoxicity or hepatotoxicity. CsA determined by a radioimmunoassay was present in the fetal circulation during gestation and maternal breast milk at similar concentrations to those in the mother. Interestingly, fetal serum at birth displayed 25% suppression of a third-party mixed lymphocyte culture compared with control incubations, adding to the body of evidence which advises against the breastfeeding of children by CSA-treated mothers [77]. Whether exposure to CsA through breast milk poses a big enough risk to outweigh the benefits of breast feeding remains an unknown topic.

## 7. Conclusion

In UTn, maintaining a healthy pregnancy is the most important marker of graft function. Monitoring of the patient and the fetus should be managed by a multidisciplinary team, including a transplant surgeon, high-risk obstetrician, and neonatologist [99]. The pregnant transplant recipient must consult frequently and well in advance of the labour process, with the neonatologist and the paediatrician so that both the patient and team are prepared for any untoward outcomes. CsA serum levels should be monitored closely with attention paid to graft function including visual inspection of the cervix. 

CsA is a widely used immunosuppressant by transplant recipients, required to maintain adequate graft and maternal survival. Its effect on the fetus and the variation of its influence upon the graft during pregnancy has been of major concern. Continuing advances and modifications in CsA therapy, coupled with an increasing number of successful pregnancy outcomes after all types of solid organ transplantation, have resulted in a greater confidence in CsA use during pregnancy. When used in combination with other immunosuppressants, CsA has demonstrated a more potent protective influence on the transplanted graft, with minimal side effects and a negligible teratogenic effect. 

Finally, it is worth highlighting that despite the theoretical risks of CsA and other types of immunosuppressants to mother and fetus, successful pregnancies are now considered the norm in transplant recipients. Importantly, successful pregnancy is defined as one without evidence of malformations in the newborn, as well as worsening of graft function (because of gestational factors or prepregnancy morbidity) in comparison to the prepregnancy graft state.

## Figures and Tables

**Table 1:** Immunosuppressant risk categories according to US Food and Drug Administration [18].

Category	Drug	Animal/human studies
A	Paracetamol	No risk in human studies

B	Corticosteroids (Prednisolone)	No risk in animal studies or Risk in animal studies but that risk not demonstrated in human studies

	Tacrolimus (Prograft)	
C	Rapamycin	Fetal risk demonstrated in animal studies but no adequate and well-controlled studies in humans. Drugs can be used if potential benefits outweigh risks
	Cyclosporine A (Neoral, Sandimmune, Gengraf)	

D	Mycophenolate Mofetil (CellCept, Myfortic)	Fetal risk demonstrated in human studies. In exceptional circumstances, drugs can be used if potential benefits outweigh risks
	Azathioprine (Imuran)	

**Table 2:** Effect of CsA on leucocytes [23].

Cell type	Effect
B lymphocyte	(i) ↓ cytokine production by T cells leading to inhibition of proliferation
	(ii) B-cell activation leading to induction of apoptosis
	(iii) Ligation of immunoglobulin leading to inhibition of proliferation

T lymphocytes	(i) ↓ in IL-2/3/4, GM-CSF, TNF-*α* expression
	(ii) ↓ levels of IL-2 resulting in ↓ production of T cells
	(iii) ↓ level of Ca^2+^-dependent exocytosis of granule-associated esterases

Granulocyte	(i) ↓ level of Ca^2+^-dependent exocytosis of granule-associated esterases
